# Qigesan reduces the motility of esophageal cancer cells via inhibiting Gas6/Axl and NF-κB expression

**DOI:** 10.1042/BSR20190850

**Published:** 2019-06-04

**Authors:** Lingyu Kong, Zhongbing Wu, Yang Zhao, Xin Lu, Huijuan Shi, Shugang Liu, Jing Li

**Affiliations:** 1 College of Integrated Chinese and Western Medicine, Hebei Medical University, Shijiazhuang, Hebei 050017, China; 2 Department of Traditional Chinese Medicine, Tumor Hospital of Hebei Province, The Fourth Hospital of Hebei Medical University, Shijiazhuang, Hebei 050011, China; 3 Department of Ecsomatics, Tangshan Maternal and Children Hospital, Tangshan, China

**Keywords:** ESCC, Gas6/Axl, Invasion, Migration, Qigesan

## Abstract

The present study is mainly to explore the mechanism that how Qigesan (QGS) affects the movement capacity of esophageal cancer (EC) cell. QGS incubates ECA109 and TE1 cell lines and detecting the motility of tumor cells by different experiments. Growth arrest-specific 6 (Gas6) and Anexelekto (Axl) were co-localized, and then detecting Gas6, Axl signaling pathway, and protein expression after QGS intervention. Similarly, Observing the signal localization and protein expression of P-phosphoinositide3-kinases (PI3K), P-AKT protein kinase B (AKT), P-nuclear factor-kappa B (NF-κB), matrix metalloproteinase-2 (MMP2), and matrix metalloproteinase-9 (MMP9). The results showed that the concentration of QGS was less than 200 ug/ml, and the cultured cells did not exceed 24 h, that no obvious cytotoxicity was observed. QGS significantly inhibited the mobility of ECA109 and TE1 cell lines in the concentration-dependent manner. In addition, QGS can regulate the Gas6/Axl pathway, inhibit the formation and localization of the Gas6/Axl complex, and reduce the protein activation of PI3K/AKT, NF-κB, MMP2, and MMP9. Experimental innovation shows that QGS can significantly slow down the mobility of EC cells by regulating the Gas6/Axl complex and downstream signaling pathways, and provides a theoretical basis for the pharmacological effects of QGS in the therapy of EC.

## Introduction

Esophageal cancer (EC) is a severely upper gastrointestinal malignant tumor, and has a high mortality rate. The onset characteristics of EC have obvious regional differences in the world [1,2]. In Western countries and the United States, about 50% of esophageal cancer is adenocarcinoma; Asian and Chinese esophageal cancer are mainly squamous cell carcinoma. China has the highest incidence and mortality of EC in the global. Cancer of the esophagus’ prevalence rate of in Linzhou of Henan Province, Cixian and Shexian counties of Henan Province is ten times higher than the world average [3,4]. Global Analysis Report in cancer survival 2000–2014 (CONCORD-3) shows 5-year age-standardized survival rate of esophageal cancer was mostly at 10–30% in developed and developing countries. And China’s survival rate is still low [5]. Esophageal squamous cell carcinoma (ESCC) is still a serious threat to human health [6]. Invasion and metastasis are the main biological characteristics of malignant tumors and that are also the root cause of high mortality in patients with esophageal cancer [7]. At present, ESCC treatment has no positive effect on invasion and migration, even with surgical resection or extensive use of systemic radiotherapy and chemotherapy, the overall EC patients’ 5-year survival rate is not optimistic. The real cause for the lack of effective method in esophageal cancer patients is the procedures of the invasion and migration of ESCC cells is very numerous and disorderly, and many mechanisms are still unclear [8–10]. Therefore, it is particularly important to investigate the regulation process of ESCC invasion and migration.

Qigesan (QGS) was created by Zhong-ling Cheng, a famous doctor during Qing Dynasty. In his book (Yi Xue Xin Wu), QGS was used to therapy ‘YeG’. EC belongs to the category of “YeGe” in Traditional Chinese Medicine (TCM) [11]. Studies have reported that QGS can better inhibit the metastasis of patients with esophageal cancer compared with other traditional Chinese compounds [11,12]. Our Province (Hebei, China) is one of the highest incidence areas of ESCC, so we can receive many patients of esophageal cancer. Studies have shown that QGS can improve the symptoms of esophageal cancer and postoperative patients, showing the trend of QGS can delay the recurrence and metastasis of esophageal cancer, [13]. In spite of this, the specific mechanisms of QGS inhibiting the invasion and migration of ESCC have not been illuminated.

Studies have shown that Gas6 and Anexelekto (Axl) are highly expressed in oral squamous cell carcinoma (OSCC) and other cancer tissues and cells and the binding of Gas6 and Axl affects cell migration and other functions [14,15]. Recent studies have shown that the Gas6/Axl complex promotes bone marrow metastasis of prostate cancer cells by inducing invasion and survival, and studies have also shown that Gas6/Axl was closely related to the invasion and metastasis of gastric cancer and non-small-cell lung cancer [16–18]. Furthermore, studies have reported that the Gas6/Axl-PI3K (phosphoinositide3-kinases)/AKT pathway promotes OSCC invasion, and the Gas6/Axl-NF-κB (nuclear factor-kappa B ) pathway enhances OSCC cell invasion/migration ability [19,20]. However, how the Gas6/Axl mediates the cell motility of ESCC is still unclear, and whether it promotes the enhancement of these signaling pathways remains to be further studied.

Our previous protein chip results showed that QGS can reduce the expression of Gas6 of esophageal cancer cells (unpublished data). It has been reported that Gas6 promotes the development of esophageal cancer, Gas6 expression is essentially the same as Axl and is maximally up-regulated in invasiveness and lymph node metastasis of esophageal cancer [21,22]. The protein level of Axl in ESCC tissues was significantly higher than normal. Most ESCC cell lines had higher Axl levels than non-cancer cells [23,24]. Our previous studies have shown that QGS can increase the expression of adhesion proteins and enhance cell adhesion, thereby inhibiting the invasion and migration of esophageal cancer cells. [25]. Therefore, we explore the effects of other mechanisms on cell invasion and migration, we hypothesize that QGS can inhibits the cell mobility of ESCC by inhibiting the Gas6 to regulating the Gas6/AXL signaling pathway, which leads to the decrease of PI3K, AKT, NF-κB, matrix metalloproteinase-2 (MMP2) and matrix metalloproteinase-9 (MMP9) signals. Study is in order to confirm that QGS can regulate Gas6/Axl and its downstream signaling pathway, thereby inhibiting cell invasion and migration, so as to provide theoretical basis for QGS as a treatment for esophageal cancer.

## Materials and methods

### Preparation of QGS

The Chinese materia medica of QGS come from Sinopharm Group Le-Ren-Tang Medicine Co., Ltd. (Shijiazhuang, Hebei, China). See Table 1 for QGS composition [25]. All plant medicines were identified accorded with TCMSP (Traditional Chinese Medicines Systems Pharmacology database, http://lsp.nwu.edu.cn/tcmspsearch.php) and The Plan List (http://www.theplantlist.org/). According to the clinical daily dose of each patient, the above prescription formulas were used to treat an adult with an average body weight of 60 kg for 3 day. All herbs are soaked in 1000 ml of distilled water for 1 h, decocted twice. The two extracts are mixed, pressurized and filtered, concentrated in a rotary evaporator, and then freeze-dried. QGS freeze-dried powder was extracted and placed in the refrigerator at −20°C, diluted with conventional medium, and used after filtered. In our experiments,1 mg lyophilized powder, about 0.1% of a person’s daily dose, selecting the intervention dose based on principles of dose use of TCM [25].

**Table 1:** The composition of Qigesan

Scientific name	Chinese name	Weight	%
Curcuma wenyujin	Yujin	75 g	15
Adenophora tetraphylla	Shashen	75 g	15
Radix Salviae Miltiorrhizae	Danshen	50 g	10
Fritillaria Thunbergii Bulbus	Zhebeimu	30 g	6
Poria	Fuling	30 g	6
Amomum villosum Lour	Sharen	30 g	6
Lotus leaf	Heye	15 g	3
Pinellia ternata	Banxia	30 g	6
Blighted wheat	Fuxiaomai	15 g	3
Radix asparagi	Tiandong	75 g	15
Dioscorea opposita Thunb	Shanyao	75 g	15
Total content		500 g	

### Reagents

Newborn Calf Serum (NCS), phosphate-buffered saline (PBS) (BI, Beit Haemek, Israel), Trypsin-EDTA Solution (Solarbio, Beijing, China). RPMI 1640 cell culture medium (Corning, Steuben County, New York, U.S.A.), Hochest, Mitotracker dye (Thermofisher Science, MA, U.S.A.). Experimental dimethyl sulfoxide (DMSO) is derived from the Sigma (# d2650, U.S.A.). Cell Counting Kit-8 (CCK-8 kit ) (Dojindo, Japan).

### Cell lines and cell culture

Experimental esophageal cancer cell line Eca109, TE1 purchased from Shanghai Institute of Biosciences Cell Resource Center, Chinese Academy of Sciences. Cell culture (10% NCS-RPMI-1640) and passage according to protocol, Incubator condition 5% CO_2_, 37°C.

### Counting Kit-8 (CCK-8) assay

The cells were digested by EDTA trypsin and counting, planking with 4 × 10^3^ cells/well (96-well). Then incubating cells by different concentrations (0, 100, and 200 pa/ml) of QGS for 24 h. Cytotoxicity was tested using. At the end of the treatment, removing the original medium, and adding the medium containing 10% CCK-8 solution to the cells, put in the incubator and continue to culture for 2 h. Measurement of cell absorbance using a microplate reader (Bio-Rad Laboratories, Hercules, CA, U.S.A.) at 450 nm. The optical density (OD) value of three wells, and the OD value of each group was statistically analyzed.

### High content imaging analysis

Cells were plated in Perkin-Elmer (U.S.A.) 96-well plates overnight. The medium were removed, and the cells were stained with Hochest and Mitotracker (Thermofisher Scientific, MA, U.S.A.) in the incubator for 30 min. Then remove the staining and incubating the cells with QGS (0, 100 and 200 μg/ml), and placed in Operetta High-Content Analysis System (Perkin-Elmer) for 24 h. The results were analyzed using Harmony image (Perkin-Elmer) analysis software. Cell migration is expressed as mean speed and effective distance, monitoring cell motility by time-lapse image sequences, calculation method using manufacturer’s protocol (Perkin-Elmer). In order to assess cell status, the settings were made: hoechst channel for the nuclear; mitotracker channel for the cellular cytoplasm.

### *In vitro* scratch assay

Assessing Eca109, TE1 Cells migration with IncuCyte 3 Cell Migration & Invasion Assays (Essen Bioscience, Hertfordshire, U.K.) according to User Manual. A scratch was then made using the WoundMaker tool (Essen Bioscience). In short, cells were plated and grown overnight. Gently wash the cells twice, and treated with QGS (0, 100, and 200 μg/ml) for 24 h. Then put the cells into the system and take pictures of the scratches at 0, 12, and 24 h (10× magnification). Evaluating Scratch closure rate by IncuCyte software, the result is expressed by relative wound density (RWD)%.

### Invasion assays

Transwell assay for cell invasion. Transwell system from BD, U.S.A. [26]. Incubating cells with QGS (0, 100, and 200 μg/ml) for 24 h. Base membrane was prepared with 40 ml of Matrigel from BD, U.S.A. Plating 1 × 10^4^ cells in upper chamber (NO Calf Serum), and adding calf serum-containing medium to the lower chamber. Cells are incubated overnight. Remove the matrigel and upper chamber cells with a cotton swab. The Transwell chamber was then put into the new plate, the upper compartment of the attached content and the lower compartment surface of the attached cells were fixed in 4% paraformaldehyde for 20 min and then coloring cells with crystal violet for 30 min. Finally, After washing with PBS, the cells were photographed under a microscope.

### Immunofluorescence staining

ECA109 and TE1 cells were planted on the coverslip, 5 × 10^4^ Cells/well, and pretreated with QGS (0, 100, and 200 μg/ml) for 24 h. Then, 4% paraformaldehyde fixed, 0.3% Triton X-100 permeablilized, 10% bovine serum albumin (BSA) blocked and incubated overnight at 4°C in the refrigerators:anti-GAS6 (Cell Signaling Technolgy; CST#8661), anti-AXL (CST; #67202), anti-MouseAXL (Abcam; ab89224), anti-P-PI3Kp85 (Tyr^607^) (AffinityBiosciences; AF3241), anti-P-AKT1 (Thr^308^) (AbclonalTechnology; AP0304), anti-P-NF-κB p65 (Ser^536^) (CST; #3033), followed by the appropriate FITC-conjugated or TRITC-conjugated secondary antibodies (KPL). Unbound antibodies were washed, and the cells were taken out and placed on slides, and cover with containing DAPI (CST). The cells were observed by Laser scanning confocal microscope (Leica, Germany).

### Western blotting analysis

The cells were seeded into cell culture flasks and incubating cell with QGS (0, 100, and 200 ug/ml) for 24 h. Cell lysate was prepared, and cells scraped. The suspension was put into the centrifuge (4°C, 10000 × ***g***, 5 min). The cells were lysed on ice for half an hour with lysis buffer, mixture is placed into centrifuge (4°C, 12000 × ***g***, 20 min), extracting supernatant and protein quantification using the bicinchoninic acid (BCA) kit from Pierce, U.S.A. SDS-PAGE electrophoresis was performed, then transferred to the membrane, polyvinylidene fluoride (PVDF) membrane (Millipore, U.S.A.). PVDF membranes were pre-blotted with 5% BSA (Sigma, U.S.A.). Incubating PVDF membranes with primary antibody overnight: anti-GAS6 (CST; #8661), anti-RabbitiAXL (CST; 67202), anti-PI3K-P85 (CST; #4292), anti-AKT1 (CST; #75692), anti-NF-κB-P65 (CST; #8242), anti-PI3K p85 (Tyr^607^) (Affinity Biosciences; AF3241), anti-P-AKT1 (Thr^308^) (AbclonalTechnology; AP0304), anti-P-NF-κB p6 5 (Ser^536^) (CST; #3033), anti-RabbitiMMP-2 (CST; #40994), anti-RabbitiMMP-9 (CST; #13667), anti-β-ACT (Abclonal Technology; AC026). The next day labeling fluorescent secondary antibody. Imaging PVDF membrane with Odyssey infrared imaging system (LI-COR Biosciences). The result is expressed by the ratio of protein/β-act.

### Statistical analysis

Experimental results were analyzed by SPSS 21.0 software. Mean ± S.D. indicates the analysis result of the data. The specific statistical method using a one-way analysis of variance (ANOVA) or unpaired two-tailed *t*-test according to the data. The *P*<0.05 means statistical difference between the experimental results.

## Results

### QGS administration concentration effect

**Figure 1:**
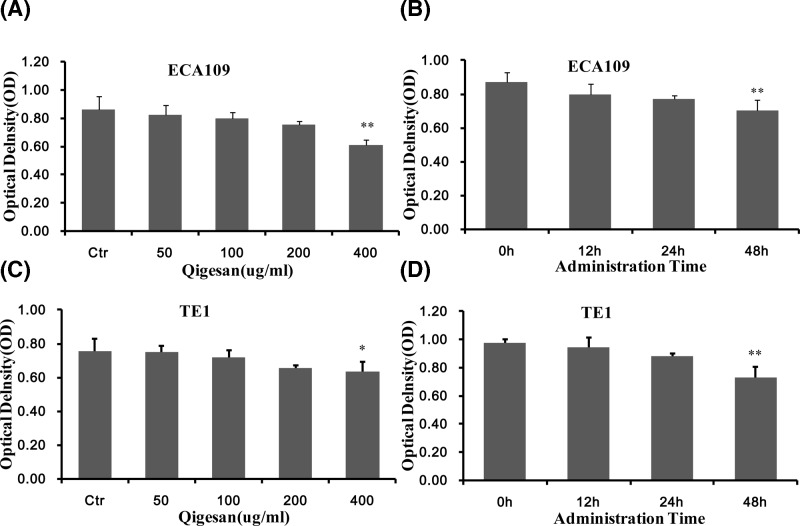
Qigesan’s administration time and effective concentration Eca109 cells (**A**) and TE1 cells (**C**) were treated with Qigesan (0, 50, 100, 200, and 400 μg/ml) for 24 h. Eca109 cells (**B**) and TE1 cells (**D**) were treated with Qigesan 200 μg/ml for (0, 12, 24, and 48 h). Cytotoxicity was quantified by OD. Combined results from three independents experiments are shown. ^*^P<0.05, ^**^P<0.01, compared with control.

CCk-8 method [27] observing the changes of Eca109 and TE1 cells in esophageal cancer after QGS intervention, and selecting reasonable administration concentration and time. We found that QGS incubated ECA109 cells for 24 h, and the concentration 50, 100, and 200 μg/ml without obvious cytotoxicity compared with the untreated group. When the concentration reached 400 μg/ml, the cytotoxic effect was significant compared with the control group. At the same time, QGS at the concentration of 200 μg/ml had no obvious cytotoxic effect within 24 h after stimulation, and it was significant at 48 h (Figure 1A,B). Similarly, we observed the same trends and results on the TE1 cells (Figure 1C,D). The present study mainly discusses the changes in the ability of esophageal cancer cells to move after QGS stimulation. Therefore, 200 μg/ml is selected as the best condition.

### QGS reduces cell migration speed and distance

**Figure 2:**
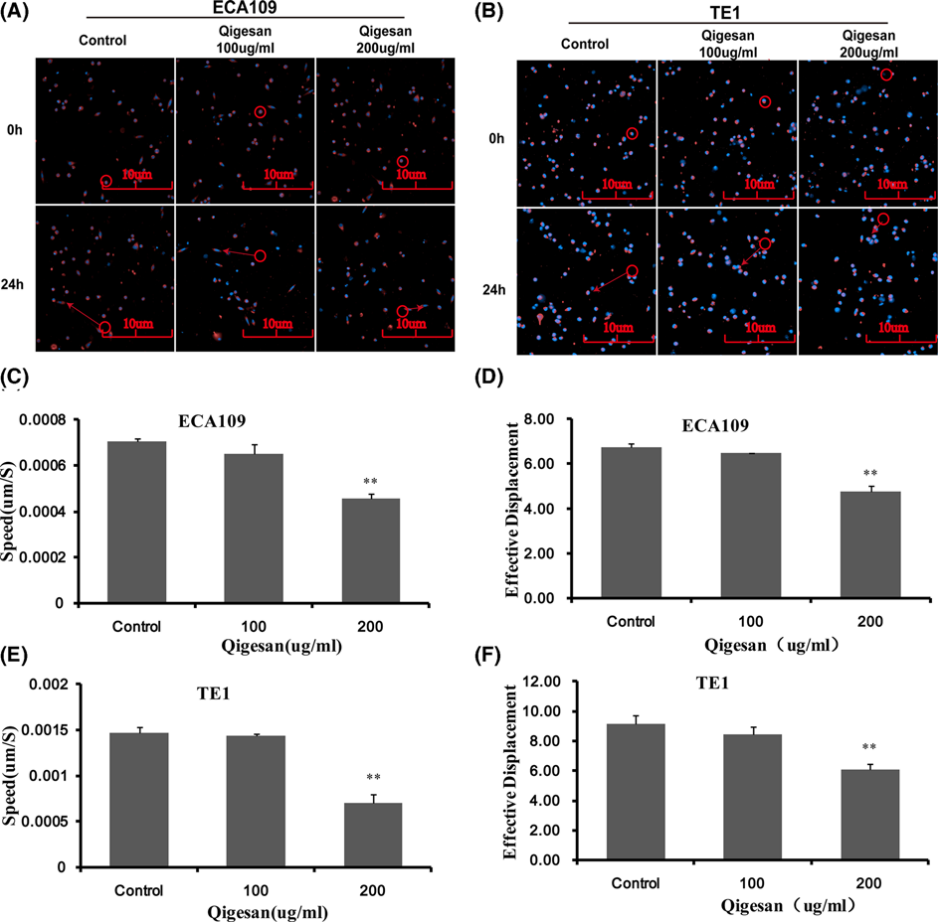
Qigesan significantly inhibitions motility and migration of Eca109 cells and TE1 cells Cells were treated with Qigesan (0, 100, and 200 μg/ml) for 24 h. (**A,B**) Representative cell fluorescence staining photographs of QGS (0, 100, 200 μg/ml) treat cells at 0, 24 h. Bar graphs showing Speed (**C,E**), unit: μm/S and effective displacement (**D,F**), unit: μm. The results are from three independent experiments. ^**^P<0.01, compared with control.

Observing the effect of QGS on migration of ECA109 and TE1 cells by Operetta CLS High content analysis system. The results showed that, in ECA109 cells, QGS showed significantly lower average movement speed of 200 μg/ml cells than the control group, and the effective movement distance of the cells was significantly shortened, while 100 μg/ml was not obviously (Figure 2A,C,D). Similarly, QGS incubates esophageal cancer TE1 cells, and we observed the same trends and results (Figure 2B,E,F).

### QGS inhibits cell migration

**Figure 3:**
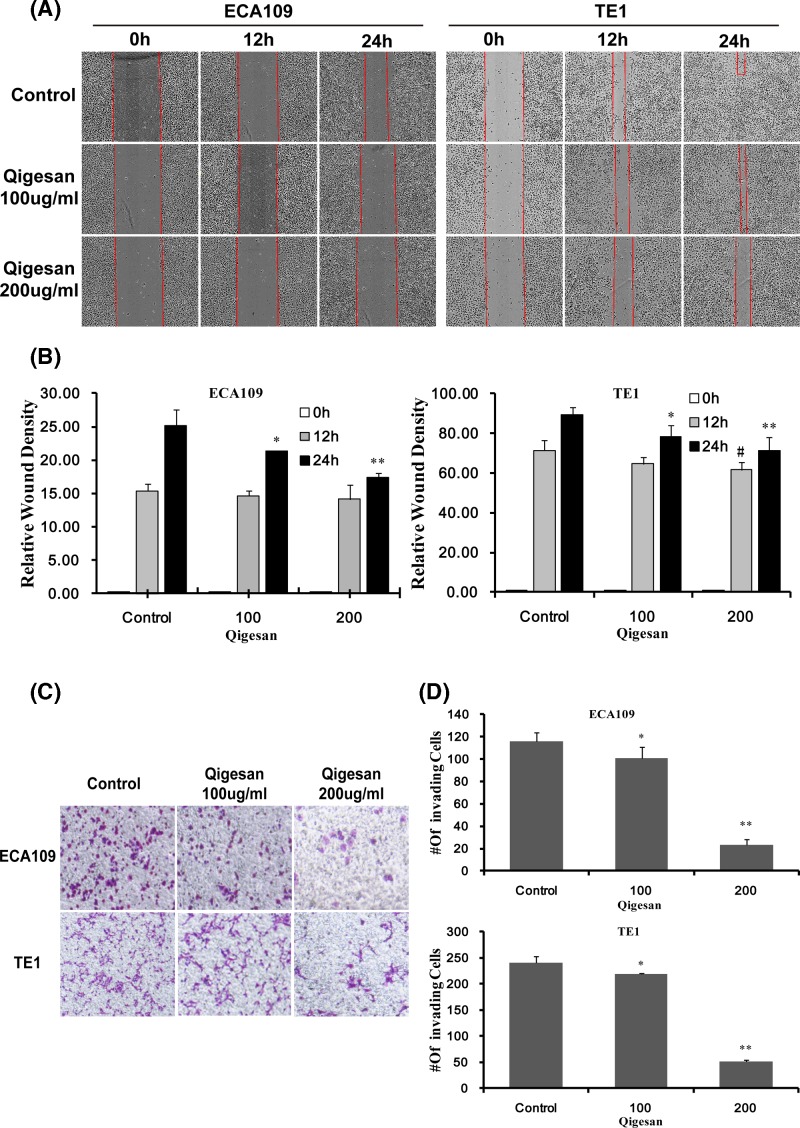
Qigesan inhibits esophageal cancer cell migration and invasion Scratch assay and Invasion assays for wound healing were performed on Eca109 and TE1 cells in the presence of QGS (0, 100, and 200 μg/ml). (**A**) Representative photomicrographs of untreated and QGS-treated cells at 0, 12, and 24 h. Red lines indicate the migrating edges of cells. (**B**) Bar graphs showing percentage RWD in treated cells, comparison with the control. The results are from three independent experiments.^ *^P<0.05, ^**^P<0.01, QGS-treated cells at 24 h compared with control; ^#^*P*<0.05, QGS-treated cells at 12 h compared with control. (**C**) Invasion assays of Eca109, and TE1 cells were performed using the Transwell system in the presence of QGS (100 and 200 μg/ml) for 24 h, and the picture on the left is Control. (**D**) Bar charts showing the number of invading cells for each cell line at doses of 0, 100, and 200 μg/ml QGS for 24 h. Combined results from three independents experiments are shown. ^*^P<0.05, ^**^P<0.01, compared with control.

To demonstrate the inhibitory effect of QGS on the migration of ESCC cell lines ECA109 and TE1, we used a scratch test. We observed that the QGS dose of 200 μg/ml group compared with the control group, which obviously inhibits cell migration in TE1 cell at 12 h. More importantly, we observed that the QGS dose of 100 and 200 μg/ml group compared with the control group, which significantly inhibits cell migration at 24 h, but the effect of 200 μg/ml was significantly better than the 100 μg/ml group, the same trend was found in both cells. And as the dose of QGS increased, the cell area growth in the scratched area was significantly inhibited correspondingly as the dose and time increase (Figure 3A,B). Therefore, these results indicate that QGS can significantly inhibit the migration of ESCC cells.

### QGS inhibits cell invasion

In order to observe whether QGS extract can affect the invasive ability of ECA109 and TE1cells, *in vitro* cell invasion experiments were carried out. We observed that the QGS dose of 100 and 200 μg/ml compared with a blank group, gradually decreased the number of cells passing through the Transwell chamber and showed a concentration-dependence, the same trend was found in both cells, but the effect of 200 g/ml was significantly better than the 100 μg/ml group (Figure 3C,D). The results showed that QGS can significantly inhibit the invasion ability of ESCC cells.

### QGS regulates Gas6/Axl signaling pathway

**Figure 4:**
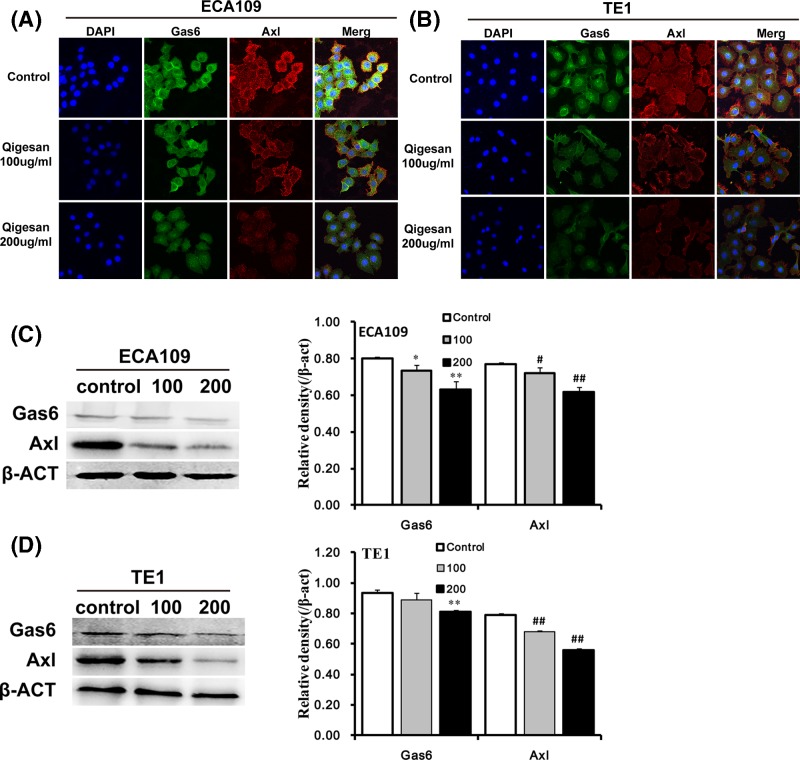
Qigesan regulates the localization and expression of Gas6/Axl Eca109 and TE1 cells were treated with QGS (0, 100, and 200 μg/ml) for 24 h. (**A,B**) Representative immunofluorescence pictures of Gas6, Axl, and Gas6/Axl channels. (**C,D**) Representative Western immunoblots of Gas6 and Axl. Bar graphs showing significant dose-dependent inhibition of relative protein density (normalized to β-ACT) of Gas6 and Axl that compared with the untreated control group. The results are from three independent experiments.^*^P<0.05, ^**^P<0.01, Gas6 relative protein density compared with control, ^#^*P*<0.05, ^##^*P*<0.01, Axl relative protein density compared with control.

To determine the QGS-affecting cell localization and expression of the Gas6/Axl complex, we used immunofluorescence and laser confocal microscopy. The results showed that the Gas6, Axl, and Gas6/Axl complex in the control group were highly expressed in the esophageal cancer cell membrane. Compared with the control, the Gas6/Axl complex was isolated in the QGS group, the expression of Gas6 and Axl was decreased and shows a concentration-dependent trend. The same trend was observed in esophageal cancers ECA109 and TE1 (Figure 4A,B). Western blotting result analysis is further presented that QGS stimulation significantly reduced the expressions of Gas6, Axl, and shows a concentration-dependent trend. The same trend was observed in both cell type (Figure 4C,D). This is proof that QGS effectively affects the Gas6/Axl signaling pathway.

### QGS inhibits PI3K/AKT and NF-κB signaling pathway

**Figure 5:**
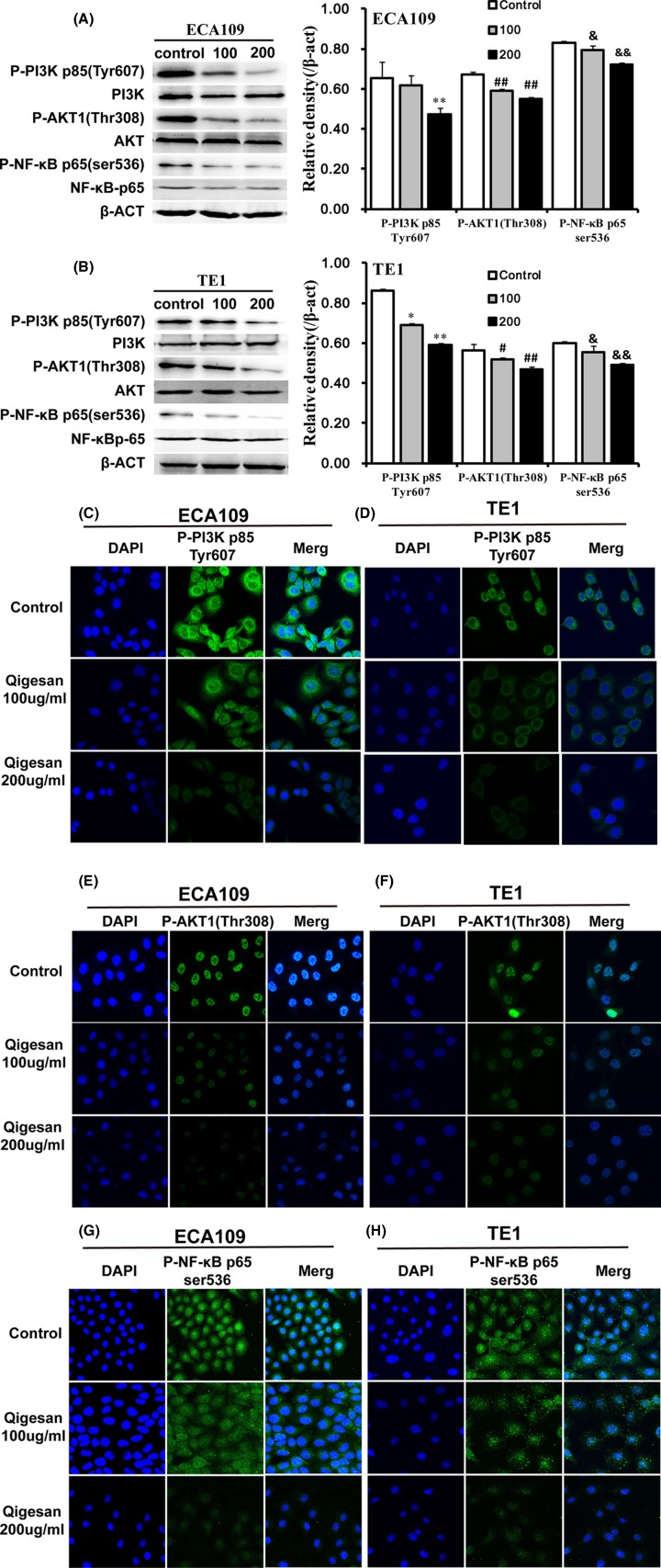
Qigesan inhibits PI3K/AKT and NF-κB signaling pathway Eca109 and TE1 cells were treated with QGS (0, 100, and 200 μg/ml) for 24 h. (**A,B**) Representative Western immunoblots of P-PI3K, PI3K, P-AKT, AKT, P-NF-ΚB, and NF-κB. Bar graphs showing significant dose-dependent inhibition of relative protein density (normalized to β-ACT) of P-PI3K p85 (Tyr^607^), P-AKT1 (Thr^308^), and P-NF-ΚB p65 (Ser^536^) that compared with the untreated control group. The results are from three independent experiments. ^*^P<0.05, ^**^P<0.01, P-PI3K-P85 (Tyr^607^) relative protein density compared with control; ^#^*P*<0.05, ^##^*P*<0.01, P-AKT1 (Thr^308^) relative protein density compared with control; ^&^*P*<0.05,^ &&^*P*<0.01, P-NF-ΚB-P65 (ser^536^) relative protein density compared with control. (**C,D,E,F,G,H**) Representative immunofluorescence pictures that P-PI3K, P-AKT, and P-NF-κB were localized. Signaling pathway were inhibited and showing significant dose-dependent.

In order to demonstrate the mechanism of QGS inhibiting mobility of ESCC cells, we studied that PI3K/AKT and NF-κB signaling pathways. Western blotting analysis results showed that QGS stimulation significantly reduced the protein expression of P-PI3K, P-AKT, and P-NF-κB and showing a trend in dose-concentration changes. Same trend of realization was discovered in esophageal cancer cells ECA109 and TE1 (Figure 5A,B). This indicates that QGS has a significant inhibitory effect on PI3K/AKT and NF-κB protein expression.

The results of immunofluorescence and confocal laser microscopy also confirmed the high expression of P-PI3k cytoplasm in the control group, as well as the high expression in the P-AKT nucleus. Compared with the control group, the QGS group significantly reduced the expression of P-PI3k, P-AKT expression and nuclear incorporation, and P-NF-κB expression and nuclear incorporation and showing a trend in dose-concentration changes (Figure 5C–H). This suggests that QGS effectively inhibits PI3K/AKT and NF-κB signaling pathways.

### QGS inhibits MMP2 and MMP9 protein pathway

**Figure 6:**
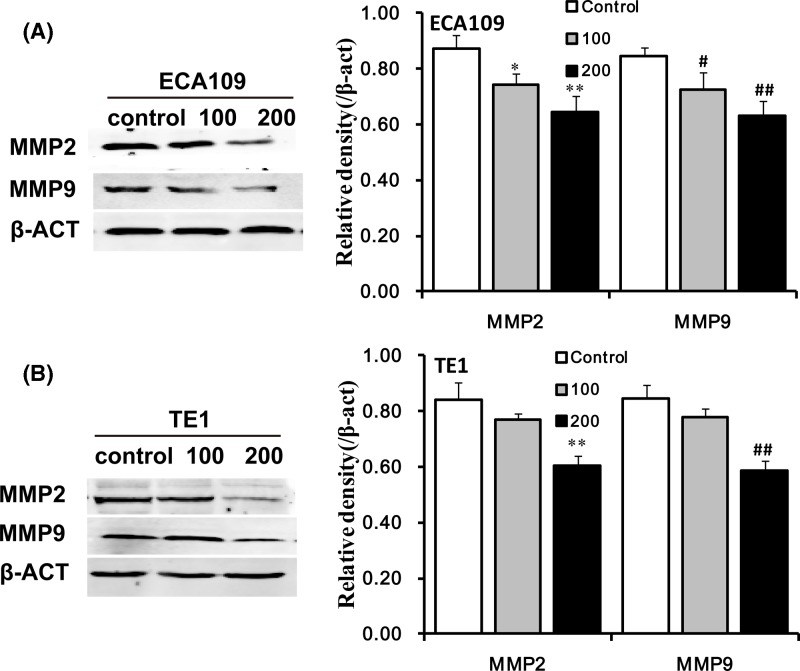
Qigesan inhibits MMP2 and MMP9 protein pathway and Mechanism diagram (**A,B**) Representative Western immunoblots of MMP2 and MMP9. Bar graphs showing significant dose-dependent inhibition of relative protein density (normalized to β-ACT) of MMP2 and MMP9 that compared with the untreated control group. The results are from three independent experiments. ^*^P<0.05, ^**^P<0.01, MMP2 relative protein density compared with control; ^#^*P*<0.05, ^##^*P*<0.01, MMP9 relative protein density compared with control.

In order to demonstrate the mechanism of QGS inhibiting cell mobility of ESCC cells, We also studied two protein pathways in which MMP2 and MMP9, these are closely related to esophageal cancer infiltration and metastasis. Western blotting analysis results showed that QGS stimulation significantly reduced the protein expression of MMP2 and MMP9 and showing a trend in dose-concentration changes. Same trend of realization was discovered in esophageal cancer cells ECA109 and TE1 (Figure 6).

## Discussion

Esophageal cancer is a severely upper gastrointestinal malignant tumor, tumor invasion, and metastasis are important factors leading to death in patients with esophageal cancer. According to the ancient Chinese book research and clinical experience, we believe that the main reason for esophageal cancer is the shortage of stomach-Yin, ‘Ganrun Ruyang’ is its fundamental treatment principle. QGS is a representative formula for ‘Ganrun Ruyang’ therapeutic guidance and has been used in China for more than 200 years. Our experience with QGS in treating esophageal cancer patients shows that QGS can improve the symptoms of dry mouth, constipation, weight loss, and other symptoms of yin deficiency in patients with esophageal cancer, after QGS treatment, the disease-free survival (DFS) of patients with esophageal cancer resection was significantly extended, and show a good trend to inhibiting metastasis [13]. In order to confirm the inhibition of QGS on invasion and migration of ESCC cells, we extracted QGS lyophilized powder. Intervention with ECA109 and TE1 cells with QGS. Experiments shows that QGS not only shortens the effective distance of esophageal cancer cell movement, but also reduces the average speed of movement, thereby significantly inhibiting the invasion and migration of tumor cells in a dose trend. In spite of that, the specific pathways through which QGS inhibits the invasion and migration of ESCC cells and has not yet elucidated. Therefore, we conducted a preliminary mechanism study.

Our previous protein chip results showed that QGS can reduce the expression of Gas6 of esophageal cancer cells (unpublished data). Studies have shown that Gas6/Axl is highly expressed in various tumor tissues and cells [14]. The Gas6/Axl complex acts to promote tumor progression by altering the functions of cell migration, proliferation, and survival. Many studies have shown that Gas6/AXL plays a big role in promoting tumor metastasis [28–30]. Gas6/AxL can induce invasion and survival during prostate cancer cell bone marrow metastasis [16], and also promotes invasion and metastasis of gastric cancer and lung cancer [17,18]. Therefore, we believe that QGS likely regulates the invasion and migration of ESCC cells through Gas6/AXL. Experiment results also demonstrate that QGS can effectively inhibit the expression of Gas6 and AXL, and can affect the cell localization of Gas6/AXL complex.

AXL is an important member of the receptor tyrosine kinases, which is activated by its ligand GAS6 and triggers downstream signaling pathways according to different cell types, such as inositol PI3K/AKT, and nuclear transcription factor NF-κ B. It can perform many functions, including affecting cell migration, cell metastasis, and cell adhesion [31–33]. Studies have shown that PI3K and AKT expression levels were significantly increased in esophageal cancer, and played a role in promoting the invasion and metastasis of esophageal cancer cells [34,35]. The present study also demonstrated that PI3K/AKT signaling pathways are activated in esophageal cancer cells TE1 and ECA109, and that AKT is mainly expressed in the nucleus, consistent with previous reports [36,37]. Similarly, the NF-κB signaling pathway also can promote the occurrence and progression of esophageal cancer. Research also demonstrated increased activation of the NF-κB signaling pathway in esophageal cancer cells TE1 and ECA109. Therefore, inhibition of PI3K/AKT and NF-κB pathways is likely to be an effective method for the treatment of ESCC [38]. Experimental results of this research demonstrate that QGS can separate Gas6/Axl and inhibit its protein expression. Gas6/AXL, as a decrease in expression of the upstream pathway of the cell membrane, which suppressing and reducing PI3K/AKT and NF-κB pathways.

**Figure 7:**
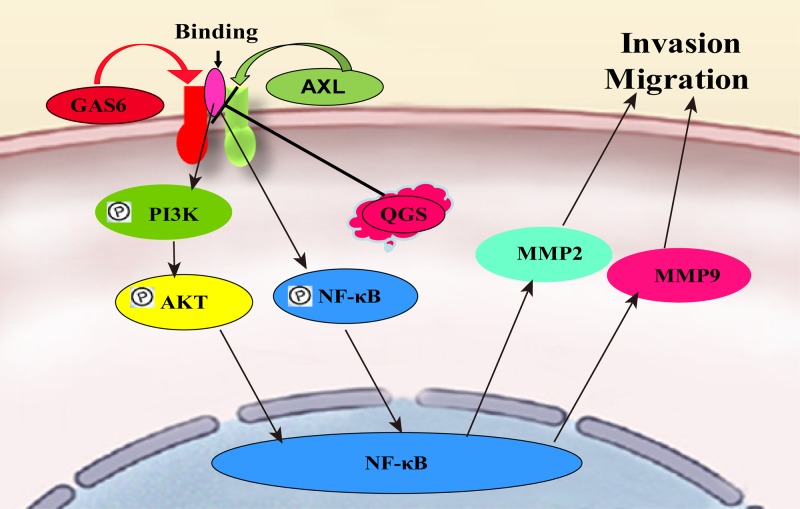
QGS regulates Gas6/Axl and downstream pathways inhibit esophageal cancer cell invasion and migration QGS regulates Gas6/Axl by inhibiting the binding of Gas6 and Axl, thus inhibits PI3K, AKT, and NF-κB pathway, and QGS decreasing expression of NF-κB nucleus, then inhibiting tumor cell invasion and migration via reduces the expression of MMP2 and MMP9.

This research is mainly to observe QGS impact on the mobility of esophageal cancer, we chose the NF-κBp65 according to the relevant literature reports [39,40]. MMP2 and MMP9 are important protein pathways for tumor invasion and metastasis. Studies have confirmed that in esophageal cancer and gastric cancer, MMP9 and MMP2 act as targets of NF-κB, which promote the invasion and metastasis of tumor cells [40–42]. Invasion and metastasis of Eca109 cells can be inhibited by inhibiting the expression of NF-κB and MMP2 [43]. Studies have shown that the block Human MMP9 NF-κB binding site can inhibit the mobility of Human Osteosarcoma Cell. MMP9, as a target of NF-κB, can function through the p65 site [44]. Experiments have shown that NF-kB binding sites: NF-κB I (−1418/−1409), NF-κB II (−626/−617), and NF-κB III (−353/−345), of which NF-κB II (−626/−617) binding to MMP9 promoter [45]. This experiment also showed that QGS inhibits the migration of esophageal cancer cells by affecting p65 phosphorylation and inhibiting the expression of MMP9. NF-κB can mediate the expression of MMP and promote the invasion of gastric and colorectal cancer. Furthermore, NF-κB promotes the invasion of EAC cells by activating MMP-2/9 [46]. Studies have shown that QGS can inhibit cell migration by inhibiting the activity of MMP2 and MMP9, and the inhibitory effect on MMP9 is greater than MMP2, which is consistent with the results obtained in this experiment [47]. The results and references in the present study confirm that GAS6/AXL is an important pathway affecting the invasion and metastasis of esophageal cancer. QGS can significantly inhibit the migration of ESCC cells by regulating the GAS6/AXL pathway by QGS, inhibiting the binding of the complex, thereby affecting the downstream PI3K/AKT and NF-κB pathways. Like the phosphorylation of YAP principle [48], NF-κB entry into the nucleus reduced, and thus inhibiting MMP2 and the expression of MMP9 reaches inhibition of tumor cell invasion and metastasis (Figure 7).

In recent years, TCM has carried out a lot of beneficial research in the treatment of cancer. TCM can improve the survival rate of patients with hepatocellular carcinoma after radiofrequency ablation [49]. Adjuctive therapy with TCM can significantly improve the clinical symptoms of liver cancer, gastric cancer patients on the basis of conventional treatment, and the proportion of distant metastasis in the TCM use was significantly lower than in non-TCM use [50,51]. TCM formula can reverses cell adhesion-mediated drug resistance in lung cancer cells [52]. Some Chinese herbal compounds have been reported to have potential anticancer effects [53]. However, there are still few studies on the anti-cancer mechanism of TCM.

In our hospital, we observed that QGS has a positive effect on controlling metastasis and improving symptoms in patients after esophageal cancer surgery [13]. Clinical observations show that QGS can significantly improve the clinical symptoms of patients with esophageal cancer, and no adverse drug reactions have been found [54]. The results of the animal experiments showed that QGS fed the experimental mice for 12 weeks and no related toxicity was found [55]. The present study demonstrates that QGS inhibits invasion and migration of ESCC cells by regulating the Gas6/AXL signaling pathway and thereby causing a decrease in PI3K/AKT and NF-κB signaling, preventing metastasis of esophageal cancer. The present study indicates that Gas6/AXL may be the main target of QGS. These results will be conducive to further research and development of Chinese herbal compounds. These results also provide clinicians with new methods to inhibit esophageal cancer. Next, we will further study the other mechanisms of QGS inhibiting esophageal cancer.

## Abbreviations

AKT: protein kinase B
Axl: anexelekto
CCK-8: cell counting kit-8
CST: cell Signaling Technolgy
EAC: esophageal Adenocarcinoma
EC: esophageal cancer
ESCC: esophageal squamous cell carcinoma
FITC: fluorescein-5-isothiocyanate
Gas6: growth arrest-specific 6
MMP2: matrix metalloproteinase-2
MMP9: matrix metalloproteinase-9
NCS: newborn calf serum
NF-κB: nuclear factor-kappa B
OD: optical density
OSCC: oral squamous cell carcinoma
PBS: phosphate-buffered saline
PI3K: phosphoinositide3-kinases
QGS: qigesan
RPMI: roswell Park Memorial Institute
RWD: relative wound density
TCM: traditional Chinese medicine
TRITC: tetramethyl rhodamine isothiocyanate
YAP: yes-associated protein
